# Asymmetric Gingival Margins of Maxillary Central Incisors: Does It Matter to Lay Persons and Professionals

**DOI:** 10.1111/ocr.70060

**Published:** 2025-11-22

**Authors:** Jadbinder Seehra, Laura Cockerham, Spyridon N. Papageorgiou, Jonathon T. Newton, Martyn T. Cobourne

**Affiliations:** ^1^ Faculty of Dentistry, Oral & Craniofacial Sciences, Centre for Craniofacial Development & Regeneration King's College London London UK; ^2^ Department of Orthodontics Guy's and St Thomas NHS Foundation Trust, Guy's Hospital London UK; ^3^ Department of Orthodontics Kings College Hospital NHS Foundation Trust London UK; ^4^ Clinic of Orthodontics and Pediatric Dentistry, University of Zurich, Center for Dental Medicine Zurich Switzerland; ^5^ Population & Patient Health Tower Wing, Guy's Hospital London UK

**Keywords:** aesthetics, asymmetry, gingival, gingival margins, maxillary central incisor, symmetry

## Abstract

**Objective:**

The aim of this study was to assess the aesthetic judgements made by lay people and professionals relating to an asymmetric maxillary central incisors' gingival margin position.

**Setting:**

Multi‐center institutional study.

**Materials and Methods:**

A high‐quality intra‐oral photograph of a previously treated case was manipulated (six images). Parents of children undergoing conventional orthodontic treatment (PCT), parents of children undergoing combined surgical‐orthodontic treatment for a unilateral impacted maxillary central incisor (PI) and professionals (dental and orthodontic specialists) were asked to evaluate and rank the images in terms of aesthetics and provide free‐text responses to support their rankings (least and most attractive). All data was analysed statistically with regressions at 5% and a thematic analysis of the free‐text responses was performed.

**Results:**

The responses from 120 participants (mean age 42.2 years; Standard Deviation [SD] 8.6 years; 62.2% female) were analysed (30/group). Image E (symmetrical gingival margins with the lateral incisor gingival margins 0.5–1.0 mm below both the gingival margin of the maxillary canine and central incisor which are at the same level in relation to each other) was ranked the most attractive (60.8%). The presence of an asymmetric gingival margin between the maxillary central incisors was rated the least attractive (Images D and F; 30.9% and 39.2%, respectively). The highest and lowest mean ranking scores (adjusted for gender) were Image E (5.12 points; 95% CI: 4.76–5.48 points) and F (1.83 points; 95% CI: 1.57–2.10 points) A similar trend was evident within groups. The PCT group scored Image D the lowest (2.17 points; 95% CI: 1.72–2.62 points). Gender influenced the ranking of the images. Themes to support the ranking of the most attractive image were related to the importance of overall symmetry. Themes to support the ranking of the least attractive image were based on overall asymmetrical (uneven) appearance (*n* = 117).

**Conclusion:**

An asymmetric gingival margin position between the maxillary central incisors negatively influences the rating/perception of attractiveness by lay persons and professionals.

## 1 Introduction

Due to their size and position, the maxillary central incisors are the dominant teeth within a patient's smile arc [1]. An aesthetically pleasing smile should include aspects of symmetry and proportion between the maxillary central incisors [2] and adequate contouring of the gingival margins [3]. From a clinical aspect, symmetry of the teeth and gingival tissues near the midline is paramount to ensure optimal aesthetics [4]. In contrast, the further the teeth and gingival tissues are away from the midline, small asymmetric variations are deemed acceptable [4]. An asymmetric smile can be a consequence of tooth size discrepancies, aberrant shape and morphology of teeth, canting of the occlusal plane and uneven vertical position of the gingival margins [5]. The latter can be an iatrogenic effect of multidisciplinary treatment to erupt unilaterally impacted anterior teeth [6].

In the ideal aesthetic arrangement, the gingival margin of maxillary central incisors should be at the same level [7]. Uneven gingival margins can negatively influence the judgement of dental aesthetics [8]. It is established that in the rating of dental attractiveness, the judgements made by lay persons and dental professionals tend to be more negative when an asymmetric gingival margin position between the maxillary central incisors is present [9, 10, 11]. However, in these studies an insight into the raters' (laypersons') perspective and rationale to support judgements is often absent, which typifies the general lack of patient‐reported outcomes in the dental and orthodontic literature [12]. Furthermore, the raters selected in these studies tend not to include individuals where an asymmetric gingival margin position could be of clinical relevance. Management strategies for children with impacted maxillary incisor teeth generally involve a multidisciplinary approach with surgical exposure and bonding of the impacted tooth or teeth, with or without space creation within the anterior maxillary arch, and post‐surgical‐orthodontic traction [13]. However, depending on the type of surgical exposure performed, there is a risk of an increased crown length following orthodontic alignment [6]. The impact of this aesthetic deficit on the patient and their caregiver as a result of this variation in the gingival margin position is unknown.

The aim of this study was to assess the aesthetic judgement made by laypersons (parents of children undergoing conventional orthodontic treatment and parents of children undergoing combined surgical‐orthodontic treatment for a unilateral impacted maxillary central incisor) and professionals (dental and orthodontic specialists) of asymmetry in maxillary central incisor gingival margin position. The objective of this study was to explore participants' reasons for the aesthetic judgements using a thematic analysis of the free‐text responses.

## 2 Materials and Methods

### 2.1 Study Design and Patient Selection

**FIGURE 1:**
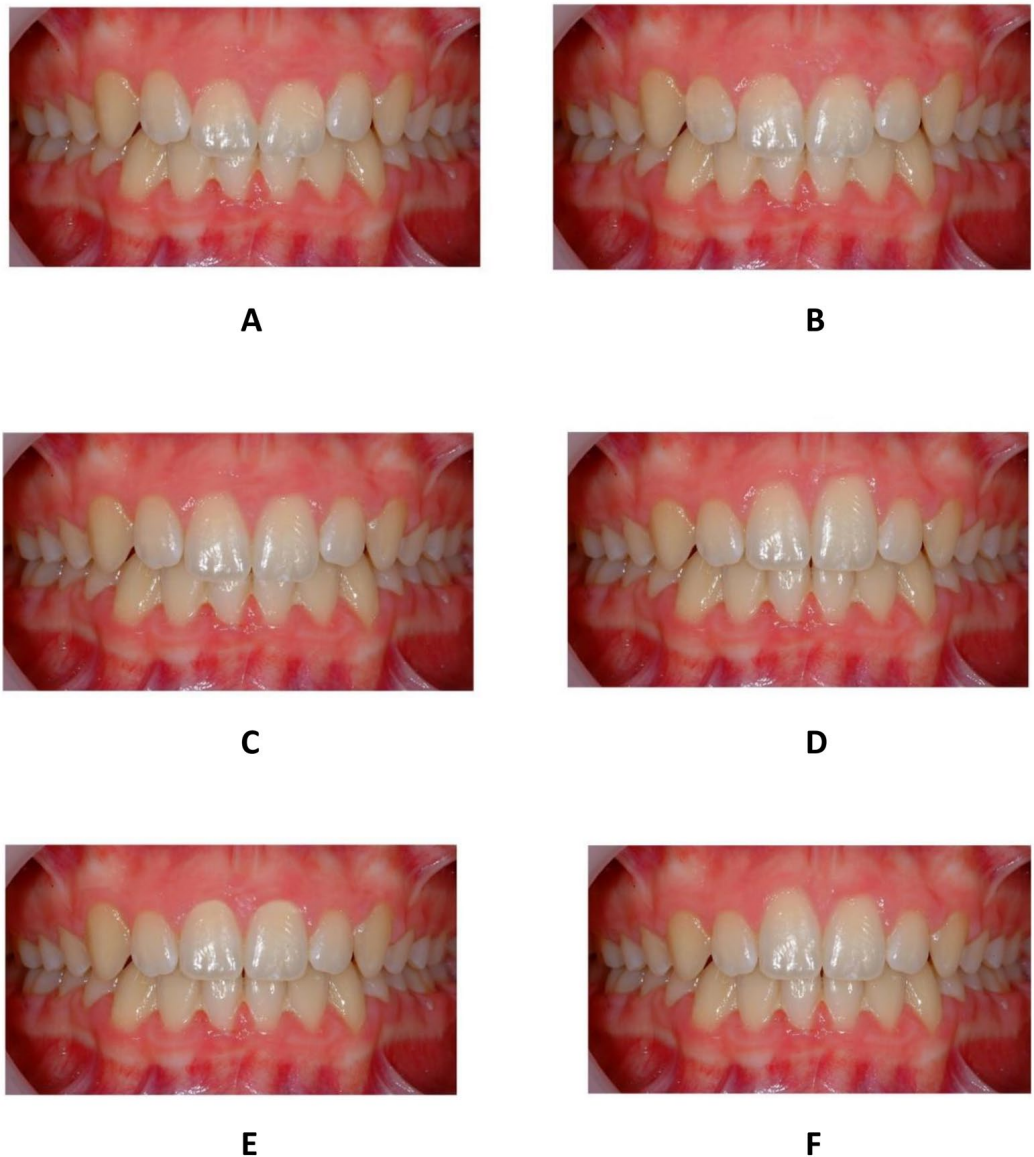
Six image manipulations based on previous research assessing aesthetic judgements (least attractive and most attractive) of dental attractiveness made by lay people, orthodontists and dental professionals.

Ethical approval for this observational study was granted by the research ethics review board at King’s College London MRSP‐22/23‐37957. A high‐quality frontal photograph of a clinical case (female patient) previously treated with fixed appliances on a non‐extraction basis, was manipulated in Adobe Photoshop 22.0 (Adobe Inc., California, USA) using the rectangular marquee and clone stamp tools, montaged onto a single A4 page and used for evaluation within a questionnaire provided to study participants. For this image manipulation, the region of interproximal contact and gingival margins of the maxillary central incisors were used as the midline vertical and horizontal reference lines, respectively. Based on previous research assessing aesthetic judgements (least attractive and most attractive) of dental aesthetics made by lay people, orthodontists and dental professionals the following six gingival margin contour variations were manipulated (Figure 1A–F): (A) gingival margin of maxillary central incisors 1.5 mm below the gingival margin of maxillary canine [10]; (B) gingival margins of upper maxillary central and lateral incisors at the same level and 0.5 mm below the gingival margins of the canines [2]; (C) gingival margins of upper maxillary central, lateral incisors and canines all at the same level [8]; (D) asymmetric gingival margin aesthetics with the upper left maxillary central incisor gingival margin 1.5 mm above the gingival margin of the maxillary canine [2, 11]; (E) symmetric gingival margin aesthetics with the lateral incisor gingival margins 0.5–1.0 mm below both the gingival margin of maxillary canine and central incisor which are at the same level in relation to each other [3, 14] and (F) asymmetric gingival margin aesthetics with the upper right maxillary central incisor gingival margin 1.5 mm above the gingival margin of the maxillary canine [2]. No further alteration in any other parameters between images, including incisal edge position, gingival color and shape, or coronal morphology was undertaken.

Parents of children undergoing conventional orthodontic treatment (no impacted teeth or multidisciplinary treatment) (PCT) with fixed appliances and parents of children undergoing combined surgical‐orthodontic treatment for a unilateral impacted maxillary central incisor (PI) at the orthodontic departments at Guys and St Thomas NHS Foundation Trust and Kings College Hospital NHS Foundation Trust were approached and invited to take part in this study. Dental and orthodontic specialists working at the same two institutions were also invited. Parents of children or adolescents currently being treated for absent teeth, trauma to the maxillary incisor teeth, or surgical correction of jaw discrepancies were excluded.

To maintain participant confidentiality, the following protocol was implemented: (1) the front cover sheet of each questionnaire contained a specific question asking whether they wished to participate or not; if the participant did not wish to participate, the incomplete questionnaire would be posted into the sealed collection box in a de‐identified manner, (2) based on demographic details, each participant was instructed to construct their own unique numerical de‐identification code, (3) questionnaires were completed in the department waiting room and posted into a secure sealed box in the reception area and (4) if a participant decided at any time point that they did not wish to participate, the de‐identification code allowed their data to be withdrawn from the dataset [15]. To eliminate potential response bias, PI and PCT were approached in the waiting room away from the clinical area and clinicians prior to a routine appointment.

### 2.2 Ranking of Images

Following informed consent, each participant was asked to evaluate the six images (A–F) and rank them in order of attractiveness (1 = most attractive to 6 = least attractive).

The image montage was printed onto high‐quality photographic paper to ensure optimal visualisation of the images. They were also asked to cite their reason(s) for assigning number 1 (most attractive) and number 6 (least attractive) as free‐text responses within the questionnaire. Each image ranking was assigned a score on a scale of 1 to 6, where a number 1 ranking would be given a score of 6 and a number 6 ranking would be a score of 1. Participant demographic details (gender, age and ethnicity) were collected.

### 2.3 Qualitative Assessment

To explore participants reasons for their aesthetic rankings (least and most attractive) a thematic analysis of the free‐text responses was conducted using an inductive coding approach [16]. This methodological framework consists of six phases (familiarising yourself with the initial data, generating initial codes, searching for themes, reviewing themes, defining and naming themes and producing the report) which helps to organise, identify, and report themes within qualitative research datasets such as free‐text responses. All six phases were conducted in sequence and independently at each stage by two assessors (JS and LC). Following completion of each phase, both assessors met to discuss the dataset. Any discrepancies in interpretation were discussed between both assessors (JS and LC) until a consensus was reached. Overlapping codes were collated, which guided the generation of initial themes. These themes were refined through discussions between both assessors (JS and LC), ensuring that each theme accurately represented the content (reasons for most attractive and least attractive choices) expressed by study participants. Themes were defined based on the frequency and significance of recurring topics across the free‐text responses. Initial themes were depicted in a thematic map, resulting to the identification of the final main themes. All data obtained in each completed questionnaire was collated in a pre‐piloted data collection sheet.

### 2.4 Statistical Analysis

Sample size calculation was based on a previous similar study [17] that reported for the highest‐ranked group an average mean ranking score of 3.88 points with a Standard Deviation (SD) of 1.90 points. Using a one‐way analysis of variance to assess a maximum difference of about one SD (moderate effect) across four groups with an equally distributed step (means of 3.88, 3.25, 2.62, and 1.98 points) and having the same SD (1.90 points), alpha of 5% and beta of 10% (power of 90%), a total of 28 participants in each group would be needed. This was increased by about 10% to 30 participants per group to account for data losses from non‐responders.

Descriptive statistics were calculated, including mean and SD for continuous variables (after checking for normality visually and with the Shapiro–Wilk test) and absolute/relative frequencies for categorical variables. Ethnicity was categorised as Caucasian or non‐Caucasian for the analyses. Differences in characteristics between participants able and not able to rank the images were assessed with Mann–Whitney or chi‐squared tests. The average ranking score (and 95% CI) was calculated for each image (to a maximum of six points with greater scores indicating better aesthetic outcome) and analysed with generalised linear models adjusting for age, gender, ethnicity, participant group and considering clustering within each participant, while pairwise comparisons were conducted using a Sidak correction for multiple comparisons. All analyses were run at an alpha of 5% in Stata (version 14.0; Stata Corp, College Station, TX) with an openly provided dataset [Zenodo. https://doi.org/10.5281/zenodo.15348888]. The protocol for this study was not registered.

## 3 Results

A total of 120 participants (30 in each group) took part in this investigation (Table 1). The mean age of the cohort was 42.2 years (SD 8.6) with a higher preponderance for females (62.2%) compared to males (37.8%). The ethnicity distribution of the sample was as follows: Caucasian (42.7%), Asian (27.3%), Black (10.9%), mixed (2.7%) and other (15.5%). In the PI group, an equal number of children were having treatment for either the unilateral impacted upper right (*n* = 15) or upper left (*n* = 15) maxillary central incisor tooth. For the entire sample (*n* = 120), the image most frequently ranked the most attractive (#1) was Image E (60.8%), followed by Image C (15.8%), Image B (15.0%), Image A (5.0%) and Image F (3.3%) (Table 2). Image D was not deemed the most attractive image by any of the participants. Conversely, the image most frequently ranked the least attractive (#6) was Image F (39.2%), followed by Image D (30.8%), Image A (27.5%), Image C (1.7%) and Image B (0.8%). Image E was not deemed the least attractive image by any of the participants (Table 2).

**TABLE 1:** Characteristics of included patients.

Variable	Category	*N* (%)
Gender (*N* = 111)	Male	42 (37.8%)
	Female	69 (62.2%)
Age (*N* = 104)	Mean (SD)	42.2 (8.6)
Ethnicity (*N* = 110)	White	47 (42.7%)
	Asian	30 (27.3%)
	Black	12 (10.9%)
	Mixed	3 (2.7%)
	Other	17 (15.5%)
	Not stated	1 (0.9%)
Group (*N* = 120)	PCT	30 (25.0%)
	Dental specialist	30 (25.0%)
	Orthodontic specialist	30 (25.0%)
	PI	30 (25.0%)

Abbreviation: SD, standard deviation.

**TABLE 2:** Rank given to each image by the responders (*n* = 120).

Position	Image A	Image B	Image C	Image D	Image E	Image F
1st most common	#4 (40.0%)	#3 (35.8%)	#2 (33.3%)	#5 (41.7%)	#1 (60.8%)	#6 (39.2%)
2nd most common	#6 (27.5%)	#2 (35.0%)	#3 (28.3%)	#6 (30.8%)	#2 (15.8%)	#5 (35.8%)
3rd most common	#3 (13.3%)	#1 (15.0%)	#4 (17.5%)	#4 (17.5%)	#3 (14.2%)	#4 (15.8%)
4th most common	#2 (8.3%)	#5 (8.3%)	#1 (15.8%)	#2 (6.7%)	#5 (5.0%)	#3 (5.0%)
5th most common	#5 (5.8%)	#4 (5.0%)	#5 (3.3%)	#3 (3.3%)	#4 (4.2%)	#1 (3.3%)
6th most common	#1 (5.0%)	#6 (0.8%)	#6 (1.7%)			

When adjusted for gender, the images achieving the highest and lowest mean ranking scores were Image E (5.12 points; 95% CI: 4.76, 5.48) and F (1.83 points; 95% CI 1.57–2.10 points) respectively (Table 3). Similarly, the highest and lowest mean ranking image scores per group were PCT (Image E; 4.77 points; 95% CI 4.32–5.21 points) and Image D (2.17 points; 95% CI 1.72–2.62 points), dental specialists (Image E; 5.77 points; 95% CI 5.31–5.83 points) and Image F (2.03 points; 95% CI 1.73–2.34 points), orthodontic specialists (Image E; 5.27 points; 95% CI 4.83–5.71 points) and Image F (1.63 points; 95% CI 1.36–1.91 points) and PI (Image E; 5.33 points; 95% CI 4.90–5.77 points) and Image F (2.07 points; 95% CI 1.65–2.49 points) (Table 4). In the adjusted model, gender had an effect on the ranking of the images (Table 5). No other characteristic (age, gender, ethnicity, participant group) influenced the ranking of images.

**TABLE 3:** Response‐based ranking of images based on crude and adjusted model.

Image	Crude	Adjusted for gender
	Mean (95% CI)	Mean (95% CI)
A	2.84 (2.58, 3.10)	2.81 (2.32, 3.30)
B	4.41 (4.21, 4.61)	4.43 (4.11, 4.75)
C	4.36 (4.15, 4.56)	4.64 (4.34, 4.95)
D	2.13 (1.94, 2.33)	2.17 (1.89, 2.45)
E	5.23 (5.03, 5.44)	5.12 (4.76, 5.48)
F	2.03 (1.82, 2.23)	1.83 (1.57, 2.10)

Ranking	Image	Image
1st	E	E
2nd	B	C
3rd	C	B
4th	A	A
5th	D	D
6th	F	F

**TABLE 4:** Response‐based ranking of images based on crude model; stratified by group.

Image	PCT	Dental specialists	Orthodontic specialist	PI
	Mean (95% CI)	Mean (95% CI)	Mean (95% CI)	Mean (95% CI)
A	3.23 (2.64, 3.83)	2.20 (1.78, 2.62)	2.77 (2.37, 3.16)	3.17 (2.61, 3.72)
B	4.20 (3.77, 4.63)	4.40 (4.05, 4.75)	4.83 (4.49, 5.17)	4.20 (3.76, 4.64)
C	4.27 (3.75, 4.78)	4.73 (4.42, 5.04)	4.53 (4.37, 4.79)	3.9 (3.45, 4.35)
D	2.17 (1.72, 2.62)	2.07 (1.74, 2.39)	1.97 (1.69, 2.24)	2.33 (1.84, 2.82)
E	4.77 (4.32, 5.21)	5.57 (5.31, 5.83)	5.27 (4.83, 5.71)	5.33 (4.90, 5.77)
F	2.37 (1.78, 2.95)	2.03 (1.73, 2.34)	1.63 (1.36, 1.91)	2.07 (1.65, 2.49)

Ranking	Image	Image	Image	Image
1st	E	E	E	E
2nd	C	C	B	B
3rd	B	B	C	C
4th	A	A	A	A
5th	F	D	D	D
6th	D	F	F	F

Abbreviations: CI, confidence interval; PCT, parents of children undergoing conventional orthodontic treatment; PI, parents of children undergoing combined surgical‐orthodontic treatment for a unilateral impacted maxillary central incisor.

**TABLE 5:** Pairwise comparisons; given as difference in means of column minus row with its 95% confidence interval; adjusted for sex (Sidak correction for multiple comparisons).

	Image A	Image B	Image C	Image D	Image E	Image F
Ref = 1		+1.56 (1.13, 1.99) *p* < 0.001	+1.57 (0.98, 2.16) *P* < 0.001	−0.68 (−1.25, −0.10) *p* = 0.008	+2.34 (1.74, 2.95) *p* < 0.001	−0.82 (−1.47, −0.17) *p* = 0.003
Ref = 2			+0.01 (−0.49, 0.51) *p* = 1.00	−2.23 (−2.76, −1.71) *p* < 0.001	+0.78 (0.29, 1.27) *p* < 0.001	−2.38 (−2.93, −1.83) *p* < 0.001
Ref = 3				−2.24 (−2.72, −1.77) *p* < 0.001	+0.77 (0.27, 1.28) *p* < 0.001	−2.39 (−2.86, −1.91) *p* < 0.001
Ref = 4					+3.02 (2.51, 3.52) *p* < 0.001	−0.14 (−0.55, 0.26) *p* = 1.00
Ref = 5						−3.16 (−3.61, −2.72) *p* < 0.001
Ref = 6						

Abbreviation: Ref, reference.

### 3.1 Thematic Analysis

A total of 117 participants provided free‐text comments to support their rankings of the most and least attractive images. As described [16], initial themes were depicted in a thematic map (Figure S1), leading to the identification of the final common themes across the four groups (Figure S2). Themes to support the ranking of the most attractive image were primarily related to the importance of overall symmetry (e.g., ‘looks the most symmetrical’, ‘proportions and symmetry are better’, ‘most symmetrical’), which included symmetry of the teeth and gingiva (e.g., ‘teeth look in proportion and symmetrical/gum height look level’, ‘most symmetrical in gingival margin, no overlap’, ‘nice gum line central incisors and canines a bit higher than lateral’, ‘gingival margin height on centrals more aesthetic’, ‘balanced gingival aesthetic’), symmetry in the size of both maxillary central incisors (e.g., ‘2 front teeth are the same length, a nice curve at gum’, ‘evenness in size of the teeth’), and alignment of the teeth (e.g., ‘seems better aligned than other set of teeth’, ‘teeth straight and all level’, ‘all teeth look straight’, ‘most aligned teeth of all’).

In contrast, themes to support the ranking of the least attractive image were primarily based on overall asymmetrical (uneven) appearance (e.g., ‘2 front teeth uneven’, ‘gingival level is not the same or centrals look too long/out of proportion’, ‘uneven teeth (front)’, looks straight, ‘although the front teeth, one looks bigger than the other. Therefore, doesn't look too attractive/pleasing’), which related to variation in the height and size of the maxillary central incisors (e.g., ‘unattractive length of central incisors’, ‘UL1 is too long and most asymmetrical with UL1’, ‘the two front teeth seem different in size’, ‘uneven size and height of top teeth’, ‘less even large front tooth on the right side’) and asymmetry of the gingival position (e.g., ‘least attractive due to gingival contour particularly around the central incisors’, ‘least attractive/uneven gingival margins’, ‘front teeth are uneven in size as gum line higher on one tooth’, ‘asymmetry of gingival heights UR1/UL1’).

## 4 Discussion

It is evident that across all four groups (two lay persons and two professionals) the presence of an asymmetric gingival margin position (Images D and F) between the maxillary central incisors negatively influenced the rating of perceived attractiveness. Image E (symmetrical gingival margin aesthetics with the lateral incisor gingival margins 0.5–1.0 mm below both the gingival margin of the maxillary canine and central incisor which are at the same level in relation to each other) was rated the most attractive by all four groups. Exploration of the study participants' free‐text responses to support their judgements (most and least attractive image), confirmed that both lay persons and professionals consider the presence of both symmetry of the gingival margins and dental proportions (size) as key determinants in the rating of attractiveness.

Previous evidence has reported that lay persons and dental professionals have different thresholds of attractiveness when rating an asymmetric gingival margin position between the maxillary central incisors. Orthodontic specialists tend to be more critical of gingival margin asymmetries than dentists and lay persons [10], with a 0.5 mm discrepancy in gingival margin position rated as unattractive [9, 11]. In contrast, for dental specialists including dentists and lay persons the threshold for unattractiveness for asymmetric maxillary central incisor gingival margins ranges between 1.0–2.0 mm [9, 10, 11]. In our study, no difference in the rating of attractiveness was evident between the four groups in this study. A possible explanation for this could be that changes in the maxillary central incisors as opposed to teeth further away from the midline are more likely to be detected particularly when the changes are asymmetric [8]. Three out of the four groups rated Image F more unattractive than Image D. This is an interesting observation given that Images D and F are mirror images of each other. The tendency of Image D being rated more unattractive than Image F, could be influenced by the rater's eye dominance which is known to influence visual perception [18]. Rater characteristics such as level of education and age can affect aesthetic judgements of the anterior teeth [19]. In the current cohort, although the direction of the effect was not substantiated, gender was the only characteristic that had an effect on the ranking of the images. Indeed, gender is known to influence perception of aesthetics with males being less critical than females [20].

A threshold discrepancy of asymmetric maxillary central incisor gingival margin position ranging between 1.0 and 2.0 mm, is deemed unattractive by lay people [11]. At this point if a patient is concerned, clinical management strategies could be considered. This may involve a multidisciplinary approach involving orthodontic mechanics to alter the vertical. position of the maxillary central incisor supplemented with periodontal/gingival recontouring [9, 21, 22]. Conversely, an asymmetric gingival margin position can occur following combined surgical exposure and commitment to orthodontic traction to erupt an impacted maxillary central incisor [6]. Based on single studies comparing exposure techniques, maxillary central incisors treated with an open surgical exposure had higher odds for an increased crown length (MD 1.37 mm) following alignment compared to incisors treated with a closed exposure [6]. The risk of this gingival impact could be relevant to the caregivers/parents of children undergoing this treatment modality and therefore it may be prudent to discuss this during the consenting process. The importance of this is further highlighted by the finding that the PI group ranked the asymmetric appearance of the gingival margins of the maxillary central incisors (Images D and F) as the least attractive and placed increased value on symmetry and proportions when providing reasons to support their attractiveness rankings.

In this investigation we used computer‐manipulated digital images, which are recognised as a validated method to assess the appearance of the smile and in particular minor alterations in dental aesthetics when assessed by both lay persons and dental professionals [23, 24]. It has been suggested that to gauge the aesthetics of the vertical position of the maxillary central incisor, both the gingival margin and incisal edge should be altered [2, 14, 25]. However, consistent with previous investigations of altered asymmetric gingival margin aesthetics of maxillary central incisors, the incisal edges were maintained at the same level [10, 11], which would be an expected outcome following a course of orthodontic treatment. This approach is further supported by the fact that compared to incisal asymmetry, dental practitioners, dental specialists and orthodontists place more value on gingival margin position when rating smile aesthetics [5].

Incremental changes to the gingival margin heights of the maxillary central incisors and comparison with the contralateral ‘control’ tooth, which are then judged is a methodology that has been commonly adopted in the literature [9, 11, 22, 26]. It is reported that as the degree of gingival margin asymmetry between both upper maxillary central incisors increases, the rating of smile aesthetics decreases [26]. Our six images were manipulated based on the findings of previously published research, investigating the attractiveness (most and least attractive) of anterior maxillary teeth as judged by panels of lay people, dentists and dental specialists [2, 3, 8, 10, 11, 14]. A range of six images has been used previously in the literature to assess dental attractiveness [17, 19]. Four of the six manipulated images represented symmetrical changes of the gingiva architecture which may have resulted in some bias towards the ranking of the asymmetric images (Images D and F). However, this was not evident in the ranking of the images by the four participant groups where a range of different rankings (most and least attractive) was evident. Furthermore, our aim was to simulate a specific treatment outcome (asymmetric gingival margin position) post‐alignment of an impacted maxillary central incisor and gauge its impact on dental attractiveness. The latter is a priori secondary outcome (assessment of gingival aesthetics) of an ongoing two‐arm Randomised Clinical Trial (ISRCTN12709966), the iMAC Trial, following the eruption (orthodontic space opening alone versus orthodontic space opening with immediate traction) of impacted maxillary central incisors due to the presence of a supernumerary tooth [27].

Previous studies lack the involvement and judgement ratings of stakeholders, where an asymmetric gingival margin position as a consequence of treatment is a potential clinical outcome. Additionally, there is an absence in the reporting of the rationale to support smile aesthetic judgements made by both lay persons and dental professionals. To address this, we included parents of children undergoing combined surgical‐orthodontic treatment to facilitate the eruption of an unilateral impacted maxillary central incisor. Parents of children undergoing orthodontic treatment acting as a proxy for lay persons in providing gingival margin aesthetic assessments has been previously validated [17]. It is interesting to note that the risk of developing asymmetric gingival margins when erupting unilateral impacted maxillary central incisors is not typically explained during the consenting process.

To gain further insight into the judgements made, study participants were asked to provide free‐text responses to support the images they ranked as most and least attractive. The use of free‐text responses can be a valuable addition to patient‐reported outcome measures often providing a deeper understanding of the data [28, 29]. The free‐text responses were subsequently analyzed using thematic analysis which provides insight into the perspective of different research participants [16, 30]. A potential disadvantage of this analysis is that, due to its flexible nature, the themes that are generated can be inconsistent [31]. To circumvent this issue and reduce subjectivity, each phase of the thematic analysis framework was undertaken independently by two assessors who met to discuss the generation of themes. Any discrepancies were discussed until a consensus was reached.

A potential limitation of this study is that we did not gauge the aesthetic judgements of children undergoing treatment for their unilaterally impacted maxillary central incisor. However, this could have introduced a degree of bias, as there are no known baseline thresholds of acceptance of smile aesthetic variations in the adolescent population [32]. Furthermore, adolescent patients undergoing orthodontic treatment tend to value health‐related behavioural changes, dental health and psychosocial benefits rather than smile aesthetics [33]. Inter‐rater reliability was not assessed as it has been reported that the assessment of smile aesthetics can be subjective [10, 11]. In contrast to previous studies which employed a visual analogue scale (VAS) method to assess smile aesthetics we used rank ordering to allow us to compare within and between groups. This was justified on the basis that VAS is known to have poor intra‐examiner reliability [34] and is subjective [35]. VAS is also more useful for assessing change within individuals rather than comparing differences across different groups [35, 36]. Furthermore, VAS allows participants to record a neutral response as opposed to ranking the order of preference (least and most attractive) [36]. To reduce further subjectivity, we used the recommendations proposed for risk of bias assessments of primary studies, where the documentation of the rationale/reasons for judgements is encouraged [37].

## 5 Limitations

It has been debated that in the assessment of smile aesthetics the image should include the full‐face [20] and lips [38] rather than just the dentition. However, numerous studies have reported that there is no difference in smile aesthetic judgements made by lay persons and dental specialists from either full‐face or close‐up dentition views [14, 38, 39, 40], in particular when assessing gingival margin asymmetries in the anterior maxillary dentition [40]. This suggests the background facial features such as eye, nose and hair do not have an influence. Additionally, lay persons are more aware of dental aesthetics when they view a close‐up image of the dentition as opposed to a full smiling face image [20]. A future quantitative assessment on this topic reporting clinically relevant estimates and identifying knowledge gaps would be beneficial to clinicians especially when similar methodologies have been utilised.

## 6 Conclusions

An asymmetric gingival margin position between the maxillary central incisors negatively influences the rating of perceived attractiveness by lay persons and professionals. Clinicians treating patients with unilaterally impacted central incisors should be aware that key stakeholders consider the presence of both symmetry of the gingival margins and dental proportions as key determinants in the rating of attractiveness.

## Author Contributions

**Jadbinder Seehra:** conceptualization, methodology, investigation, writing – original draft preparation and supervision and writing – reviewing and editing; **Laura Cockerham:** investigation and writing – reviewing and editing; **Spyridon N. Papageorgiou:** methodology, investigation, writing – original draft preparation and writing – reviewing and editing; **Jonathon T. Newton:** writing – original draft preparation and writing – reviewing and editing; **Martyn T. Cobourne:** methodology and writing – original draft preparation and writing – reviewing and editing.

## Ethics Statement

The study protocol was approved by the Ethics Committee of the university (reference number: MRSP‐22/23‐37957).

## Consent

Informed consent was obtained from all study participants.

## Conflicts of Interest

The authors declare no conflicts of interest.

## Supporting information


**Figure S1:** Thematic map showing initial themes.

**Figure S2:** Thematic map showing final main themes.

## Data Availability

Data are available in a repository and can be accessed via a DOI link [Zenodo: https://doi.org/10.5281/zenodo.15348888].
