# Supplementary figures and images for: Asymmetric Gingival Margins of Maxillary Central Incisors: Does It Matter to Lay Persons and Professionals
Source: Orthod Craniofac Res. 2025 Nov 22;29(1):156–64. doi: 10.1111/ocr.70060 (PMC12779231; doi:10.1111/ocr.70060)

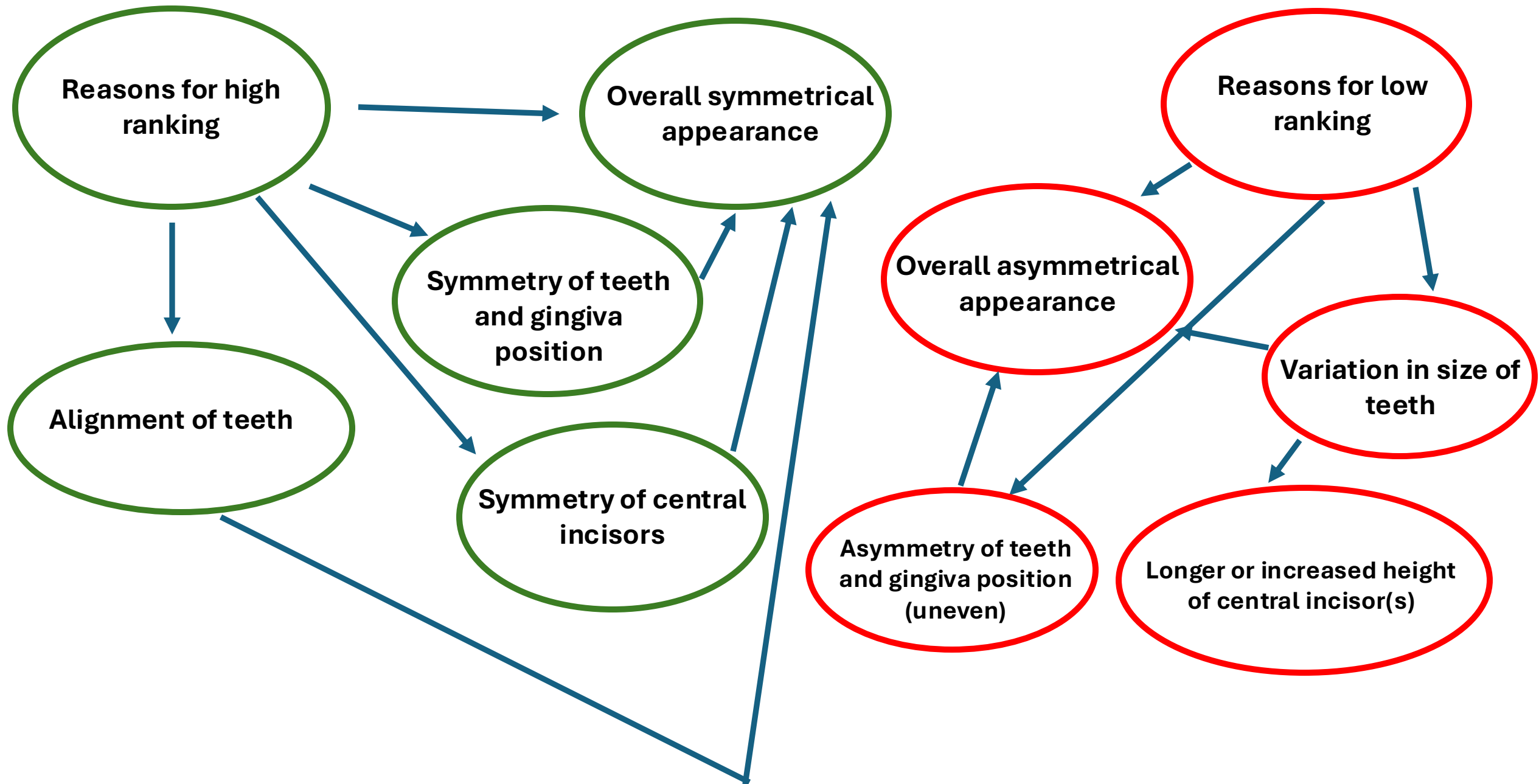

Supplement: Supplementary file 1 — Figure S1: Thematic map showing initial themes. [file OCR-29-156-s002.pdf]

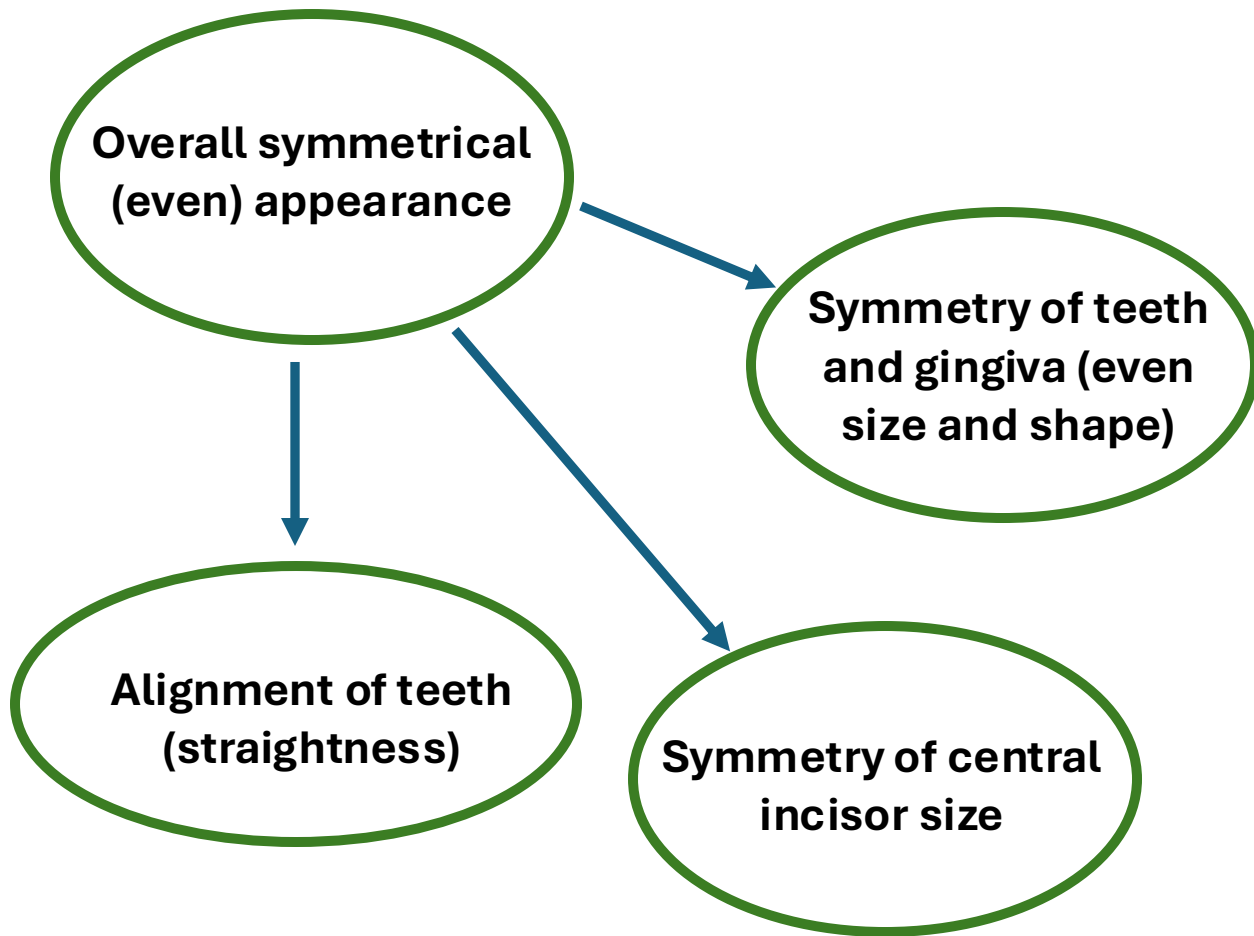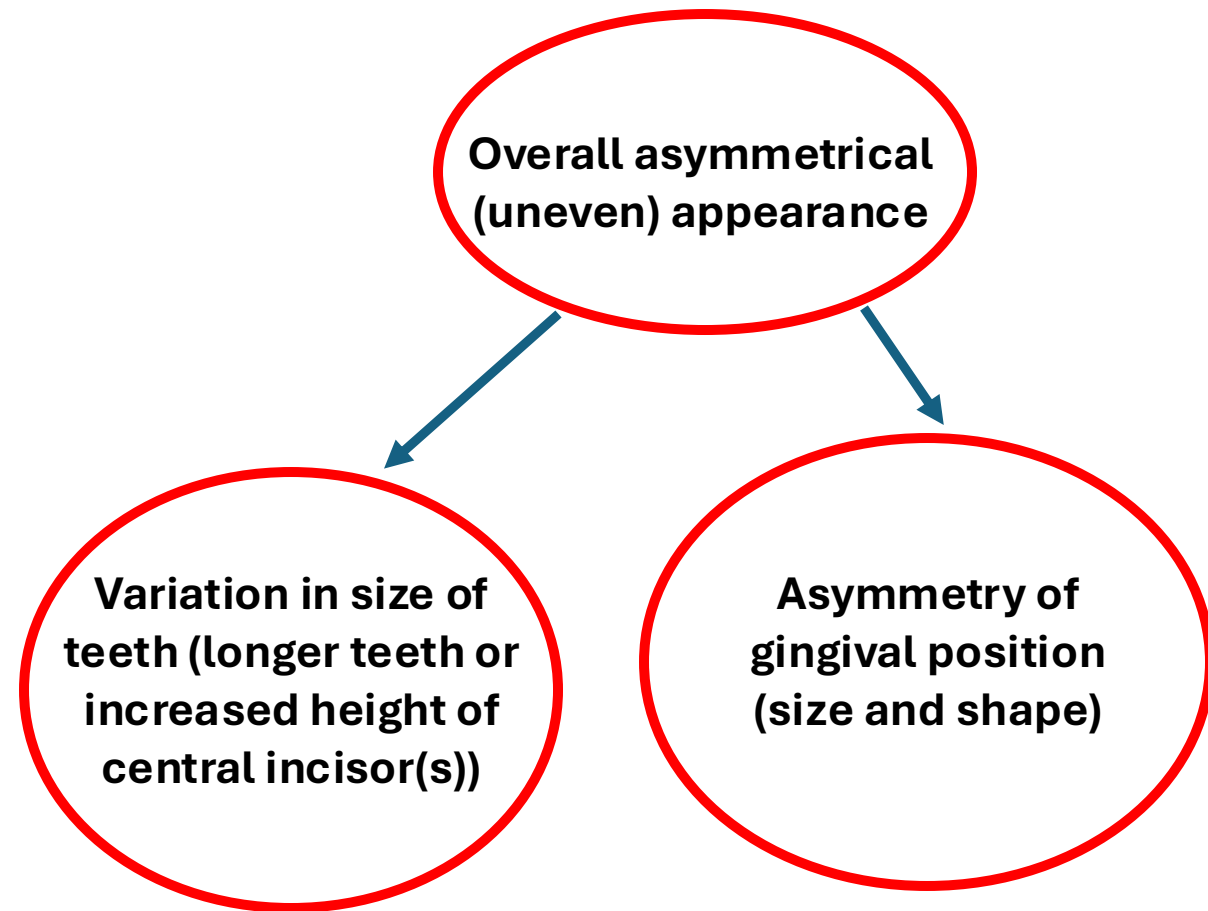

Supplement: Supplementary file 2 — Figure S2: Thematic map showing final main themes. [file OCR-29-156-s001.pdf]
